# Clinical Notes on Gonorrheal Cases Treated with Serum

**Published:** 1909-03

**Authors:** Melvin S. Rosenthal

**Affiliations:** Associate Professor of Genito-Urinary Diseases and Dermatology, College of Physicians and Surgeons, Baltimore, Maryland


### CLINICAL NOTES ON GONORRHEAL CASES TREATED WITH SERUM.

BY MELVIN S. ROSENTHAL, M. D., ASSOCIATE PROFESSOR OF GENITO-URINARY DISEASES AND DERMATOLOGY, COLLEGE OF PHYSICIANS AND SURGEONS, BALTIMORE, MARYLAND.

While a large number of cases of gonorrhea are successfully treated in a comparatively short time without complications, it also happens that quite a large percentage of all cases are followed by complications such as prostatitis, epididymitis, posterior urethritis and arthritis. All of these complications are stubborn and prone to successfully resist all forms of treatment. Arthritic cases especially are difficult to treat successfully and many of these patients become crippled or bed-ridden. Any new treatment which is based upon rational theories and appears to hold out hopes of successful treatment, merits consideration.

Rogers and Torrey call the attention of the profession to Antigonococcic Serum in articles appearing in the Journal of the A. M. A., for January 27, 1906 and September 14, 1907. This serum is prepared from the blood of rams that have been treated with gradually increasing doses of both dead and live cultures of virulent strains of gonococci. The rams are treated until they are immune and the process of preparing the serum from their blood is essentially the same as that of preparing antidiphtheric and antitetanic serums. A quantity of this serum, which was placed in my hands for clinical experimentation, I have used in the treatment of 18 cases on male patients; four private patients and 14 in the Dispensary of the College of Physicians and Surgeons. The detailed histories of these cases would be of no particular interest, but as a contribution to the literature on the subject I offer the following resume of the results of the clinical study of this serum:

There were three cases of posterior urethritis which received a total of 12 injections with the result that one was promptly cured, one showed considerable improvement and one was unimproved.

Four cases of epididymitis received a total of 10 injections; one was cured, two improved and one unimproved.

Four cases of prostatitis received a total of 7 injections; 2 were decidedly improved and 2 unimproved.

Seven cases of gonorrheal rheumatism received a total of 22 injections; 2 were cured; 2 were improved and 3 unimproved.

The dose given at each injection was the contents of one bulb of 2 ccs. of serum. This was repeated at intervals of one to four days, controlled by the clinical symptoms. The injection was made deep into the muscular tissues.

A recapitulation of the 18 cases treated is as follows:

Four (or 22 1-2 per cent.) were cured.

Seven (or 38 1-2 per cent.) were improved.

Seven (or 38 1-2 per cent.) were unimproved.

718 North Howard street.

